# Increase in Antiretroviral Therapy Enrollment Among Persons with HIV Infection During the Lusaka HIV Treatment Surge — Lusaka Province, Zambia, January 2018–June 2019

**DOI:** 10.15585/mmwr.mm6931a4

**Published:** 2020-08-07

**Authors:** Mary Adetinuke Boyd, Minesh Shah, Danielle T. Barradas, Michael Herce, Lloyd B. Mulenga, Mwansa Lumpa, Susan Ishimbulo, Ahmed Saadani, Maybin Mumba, Idongesit Essiet-Gibson, Leigh Tally, Peter Minchella, Nzali Kancheya, Annie Mwila, Khozya Zyambo, Chalilwe Chungu, Shula Chanda, Wilson Mbewe, Isaac Zulu, Terence Siansalama, Keith Mweebo, Kennedy Nkwemu, James Simpungwe, Amy Medley, Izukanji Sikazwe, Consity Mwale, Simon Agolory, Tedd Ellerbrock

**Affiliations:** ^1^CDC, Lusaka, Zambia; ^2^Division of Global HIV and Tuberculosis, Center for Global Health, CDC; ^3^Centre for Infectious Disease Research in Zambia, Lusaka, Zambia; ^4^Ministry of Health, Lusaka, Zambia.

Within Zambia, a landlocked country in southern-central Africa, the highest prevalence of human immunodeficiency virus (HIV) infection is in Lusaka Province (population 3.2 million), where approximately 340,000 persons are estimated to be infected (*1*). The 2016 Zambia Population-based HIV Impact Assessment (ZAMPHIA) estimated the adult HIV prevalence in Lusaka Province to be 15.7%, with a 62.7% viral load suppression rate (HIV-1 RNA <1,000 copies/mL) (*2*). ZAMPHIA results highlighted remaining treatment gaps in Zambia overall and by subpopulation. In January 2018, Zambia launched the Lusaka Province HIV Treatment Surge (Surge project) to increase enrollment of persons with HIV infection onto antiretroviral therapy (ART). The Zambia Ministry of Health (MoH), CDC, and partners analyzed the U.S. President’s Emergency Plan for AIDS Relief (PEPFAR) Monitoring and Evaluation Reporting data set to assess the effectiveness of the first 18 months of the Surge project (January 2018–June 2019). During this period, approximately 100,000 persons with positive test results for HIV began ART. These new ART clients were more likely to be persons aged 15–24 years. In addition, the number of persons with documented viral load suppression doubled from 66,109 to 134,046. Lessons learned from the Surge project, including collaborative leadership, efforts to improve facility-level performance, and innovative strategies to disseminate successful practices, could increase HIV treatment rates in other high-prevalence settings.

Since 2004, the U.S. government, through PEPFAR, has partnered with the government of Zambia through the MoH and National HIV/AIDS/STI/TB Council (NAC) in coordinating a national HIV response. At the time of the launch of the Surge project (January 2018), PEPFAR was supporting approximately 750,000 Zambians receiving ART, and HIV incidence rates had decreased by approximately 40% from 2004 to 2018 (*1*). The ZAMPHIA results identified remaining challenges in Zambia overall and by subpopulation in achieving the Joint United Nations Programme on HIV/AIDS (UNAIDS) 90/90/90 HIV treatment targets (90% of persons living with HIV infections know their diagnosis, 90% of those with diagnosed HIV infection on ART, and 90% of those on ART achieving viral suppression). For example, HIV treatment coverage nationally among persons aged 15–24 years was lower than that among adults aged ≥25 years. In response, the Zambia MoH, PEPFAR-Zambia, CDC-Zambia, and implementing partners started the Surge project in January 2018, with ongoing implementation coordinated via monthly reviews, quarterly leadership meetings, and joint facility site visits. Among 201 ART facilities in Lusaka Province, the Surge project prioritized 11 high-volume facilities that served 43% of persons with positive test results for HIV; each facility had annual, monthly, and weekly testing, treatment, and viral load targets. Best practices were disseminated to the other 190 ART facilities in the province through ad hoc trainings and staff member exchanges. The capacity of the HIV workforce was strengthened through HIV clinical mentors and Project Extension for Community Healthcare Outcomes (*3*), which links HIV treatment experts with distant HIV workforce staff members through video teleconference. Best practices identified from the literature and clinical practice were measured by site-level process indicators, including 1) HIV case finding via risk screening and elicitation of sexual partners from clients with a newly diagnosed HIV infection; 2) improvement in treatment initiation and retention by returning to care those clients who had missed appointments and by enrolling eligible clients into differentiated service delivery models (*4*); 3) increased documentation of viral load suppression using electronic medical record queries to identify clients with positive test results for HIV eligible for viral load testing; and 4) providing enhanced adherence counseling for clients who have not achieved viral load suppression. The Surge project emphasized routine data review and quality improvement (*5*) to identify and address gaps in HIV service delivery.

MOH, CDC, and partners routinely assessed the effectiveness of the Surge project by analyzing program data reported to PEPFAR by the 201 Lusaka Province ART facilities at the beginning of the project (January 2018) and after 18 months (June 2019). The PEPFAR Monitoring and Evaluation Report data set (*6*) contains aggregate program data and was analyzed to ascertain the number of persons with positive test results for HIV receiving ART; data were disaggregated by sex and age. Viral load suppression rate, defined as the percentage of persons receiving ART who had a documented viral load result <1,000 copies/mL within the last 12 months among all those who were eligible for viral load testing, was assessed to determine the effectiveness of ART. Quarterly numbers of persons who newly initiated ART were analyzed, including data from the 2 years preceding the Surge project, for comparison.

In the final quarter of 2018, the Monitoring and Evaluation Report definition for persons with positive test results for HIV currently receiving ART was changed from any clinical or pharmacy visit within 90 days to any visit within 30 days. This change necessitated additional analysis of 34 public ART facilities in Lusaka Province supported by the Centre for Infectious Disease Research in Zambia (CIDRZ), which had deidentified individual electronic health records that were retrospectively queried using a consistent 30-day threshold to determine the number of persons with positive test results for HIV currently receiving ART. These 34 CIDRZ-supported facilities (17% of all HIV facilities in Lusaka Province) provided treatment for 57% of persons with positive test results for HIV receiving ART. Analyses were completed using SAS (version 9.4; SAS Institute); p-values <0.05 were considered statistically significant.

In January 2018, a total of 204,091 persons with positive test results for HIV were receiving ART in Lusaka Province (based on the 90-day threshold) (Table). By June 2019, after 18 months, 103,236 persons with positive test results for HIV had newly initiated ART. The number of persons with positive test results for HIV who newly initiated ART in Lusaka Province during the Surge project was higher in each quarter (15,752–19,003 per quarter) than in any single quarter during the preceding 2 calendar years (Figure 1).

**TABLE:** Demographic characteristics and viral load suppression rates of persons with human immunodeficiency virus (HIV) infection who were currently receiving and who newly initiated antiretroviral therapy (ART),* at baseline (January 2018) and 18 months after initiation of the Lusaka Province HIV Treatment Surge (June 2019) — Lusaka Province, Zambia

Characteristic	No. (%)^†^		
	Receiving ART, January 2018^§^	Newly initiated on ART, January 2018–June 2019	Receiving ART, June 2019^¶^
**Sex (age group, yrs)^¶^**			
**Total, all ages**	**204,091 (100)**	**103,236 (100)**	**248,002 (100)**
**Male and female (<15)**	10,193 (5)	5,545 (5)	10,841 (4)
**Total female (≥15)**	**125,000 (61)**	**60,355 (58)**	**149,792 (60)**
Female (15–24)	11,342 (6)	16,192 (16)	14,544 (6)
Women (25–49)	96,736 (47)	40,935 (40)	114,469 (46)
Women (≥50)	16,922 (8)	3,228 (3)	20,779 (8)
**Total male (≥15)**	**68,898 (34)**	**37,336 (36)**	**87,369 (35)**
Male (15–24)	4,071 (2)	4,235 (4)	5,722 (2)
Men (25–49)	51,129 (25)	29,976 (29)	64,458 (26)
Men (≥50)	13,698 (7)	3,125 (3)	17,189 (7)
Viral load eligible**	192,950 (95)	—	224,764 (91)
Results documented	72,142 (37)	—	146,532 (65)
Suppressed (<1,000 copies/mL)	66,109 (92)	—	134,046 (91)

* At any of 201 Lusaka Province ART facilities.

^†^ Column values might not sum to 100% because of rounding.

^§^ Receipt of medical or pharmacy services within the previous 90 days.

^¶^ Receipt of medical or pharmacy services within the previous 30 days.

** Receiving ART for at least 6 months.

**FIGURE 1:**
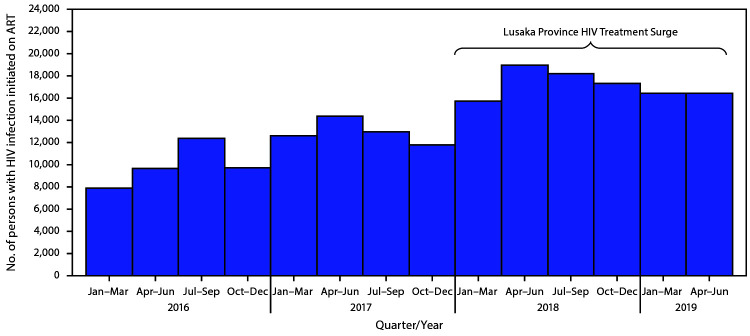
Persons with human immunodeficiency virus (HIV) infection initiating antiretroviral therapy (ART), by quarter before (January 2016–December 2017) and during (January 2018–June 2019) the Lusaka Province HIV Treatment Surge — Lusaka Province, Zambia

Compared with the percentage of persons with positive test results for HIV who were receiving ART in January 2018, a significantly higher percentage of females aged 15–24 years, males aged 15–24 years, and men aged 25–49 years initiated ART during the Surge project (16% versus 6%, 4% versus 2%, and 29% versus 25%, respectively) (p<0.001, age- and sex-disaggregated). In June 2019, using the 30-day threshold, 248,002 clients with positive test results for HIV were receiving ART in Lusaka Province, a net increase of 43,911. The age and sex distribution of clients with positive test results for HIV receiving ART in January 2018 was similar to that in June 2019. The percentage of eligible persons who had a viral load test within the preceding 12 months increased from 37% in January 2018 to 65% in June 2019; approximately 90% of clients receiving ART who had a viral load test were virally suppressed at both time points (92% in January 2018 and 91% in June 2019).

Among the subset of 34 public ART facilities with electronic health records, the number of persons with positive test results for HIV currently receiving ART (using a 30-day threshold consistently) steadily increased from 119,239 in January 2018 to 141,164 in June 2019 (Figure 2). 

**FIGURE 2:**
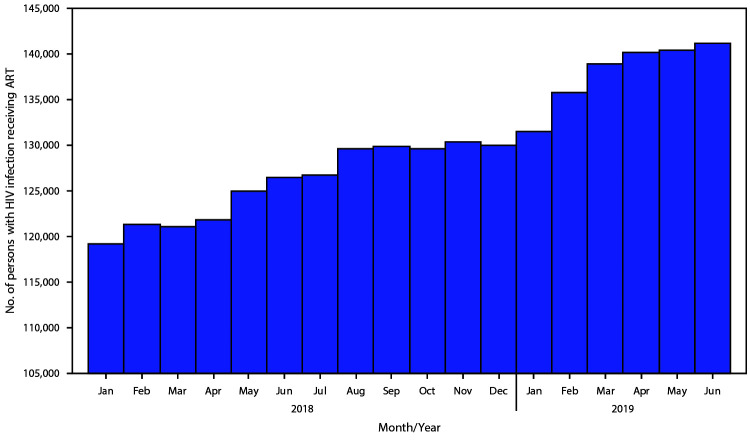
Persons with human immunodeficiency virus (HIV) infection receiving antiretroviral therapy (ART) during the Lusaka Province HIV Treatment Surge — 34 facilities with electronic health records, Lusaka Province, Zambia, January 2018–June 2019

## Discussion

During the first 18 months of the Surge project (January 2018–June 2019), approximately 100,000 persons with positive test results for HIV newly initiated ART. These new ART clients were more likely to be persons aged 15–24 years, the group that the 2016 ZAMPHIA had documented to have the lowest ART coverage. After 18 months, the number of persons with positive test results for HIV receiving ART in Lusaka Province increased from 204,000 to 248,000 (increase of 22%), and the number of individuals with documented viral load suppression increased from 66,109 to 134,046 (increase of 100%). The increase in viral load testing within the previous 12 months among persons eligible to receive testing provides a more representative estimate of viral load suppression rates at the population level than was available before the Surge project, when only a small subset of clients with positive test results for HIV who were on treatment had viral load results. Clinically, the availability of viral load results helps identify clients with positive test results for HIV with viral load suppression who would benefit from decentralized HIV treatment and ART dispensation over multiple months and clients with positive test results for HIV with unsuppressed viral loads who would benefit from targeted enhanced adherence counseling, ART regimen switches, patient support interventions, and index testing services for partners with an elevated risk for HIV exposure.

The findings from this analysis provide guidance for the next steps for the Lusaka Surge project. The gap between the number of persons with positive test results for HIV who newly initiated ART and the net change in the number currently receiving ART suggests that ART retention is a notable challenge. In addition, preliminary investigations suggest that clients who are already receiving ART might be retesting and therefore be categorized as newly initiating ART even though they are already receiving treatment. In December 2018 and January 2019, among 612 patients at 12 of the largest ART facilities in Lusaka Province with newly diagnosed HIV infection, 24% were already virally suppressed at diagnosis (unpublished data, CDC-Zambia, 2019).

Despite the success in initiating persons aged 15–24 years on ART during the Surge project, the age distribution of patients currently receiving ART in June 2019 did not appreciably change during the project. These data suggest that retention is especially challenging for this age group, a finding consistent with analyses of global HIV programs, and attributed to a variety of factors, including stigma and discrimination, mobility, and self-perceived risk (*7*). Lessons learned from implementation of the Lusaka Surge project, such as the importance of frequent coordinated data review and site visits with important stakeholders, and a focus on continuous quality improvement, can be applied to improving ART retention.

The findings in this report are subject to at least three limitations. First, HIV program data are subject to variability in reporting quality and completeness. However, this limitation is mitigated by the province-wide data quality assessment in 2017, which found high concordance between facility records and Monitoring and Evaluation Report reports, and ongoing data quality improvement efforts between CDC-Zambia, CIDRZ, and MoH. Second, the results presented here do not allow for population-level estimates of ART coverage, which are useful in assessing progress toward the 90/90/90 HIV treatment targets. In addition to ART facilities reporting to PEPFAR, private health facilities provide ART to an unknown number of clients with positive test results for HIV, which might lead to underestimation of ART coverage. Finally, estimates of HIV prevalence for Lusaka Province are subject to rapidly changing population migration and HIV risk behaviors. A second ZAMPHIA will be conducted in 2020 to inform these population-level estimates.

The Lusaka Surge project has demonstrated that collaborative leadership, political will, emphasis on improving facility-level performance, routine monitoring for accountability, and creative strategies to disseminate successful practices can increase HIV treatment rates in a high-prevalence setting. This approach can be used to target programmatic gaps that remain, including retention. With lessons learned from the Lusaka Surge project, Zambia has launched similar surges in other high-prevalence provinces with the objective of achieving 90/90/90 targets nationwide by 2020.

**Summary**

**What is already known about this topic?**

Antiretroviral therapy (ART) coverage rates among persons living with human immunodeficiency virus (HIV) infection have increased in Zambia since 2004; however, remaining gaps in coverage and viral load suppression require new strategies to achieve treatment targets.

**What is added by this report?**

An 18-month analysis of a Lusaka Province, Zambia project to increase enrollment of persons with HIV infection into ART found that the number receiving ART in Lusaka Province increased from 204,000 to 248,000 (increase of 22%), and the number of persons with documented viral load suppression increased from 66,109 to 134,046, exceeding historical performance.

**What are the implications for public health practice?**

A strategy to improve HIV treatment programs based on stakeholder coordination, frequent site visits and data monitoring, and continual quality improvement resulted in demonstrable improvements in program indicators and could be replicated in other program areas and populations.
